# The Religious Press and Quacks

**Published:** 1902-12

**Authors:** 


### EDITORIAL NOTES AND COMMENTS.

The Business office of THE JOURNAL-RECORD is No. 64 Marietta Street.

The Editorial office is 318-319-320 The Prudential.

Address all Business communications to Dr. M. B. Hutchins, Mgr.

Make remittances payable to THE ATLANTA JOURNAL-RECORD OF MEDICINE.

On matters pertaining to the Editorial and Original Communications address Dr. Bernard Wolff, Atlanta.

Reprints of original articles will be furnished at cost price. Requests for the same should always be made *on the manuscript.*

We will present, post-paid, on request, to each contributor of an original article, twenty (20) marked copies of THE JOURNAL-RECORD containing such article.

### THE RELIGIOUS PRESS AND QUACKS.

IT is evident that quacks find a fertile field for the exploitation of their wares in the religious press. In looking over a sixteen-page weekly church paper no less than forty quack advertisements were found, which included the insinuating reading notice fitted into some convenient lacuna, as well as the half-page double column with portrait of the ready testimonial writer with a few biographical data of a pathological character. The ground of human ills was pretty well covered. It is a disagreeable distortion of harmony that instruction and admonition of virtue and truth should be forced into relationship with the dishonest and mendacious claims of charlatanry, and that the same eye that seeks the Golden Text should fall upon Golden Seal. A spirit of credulousness and simple-mindedness on the part of readers of religious papers is argued that but dimly differentiates them from fools. There is an implied assumption that the mind which believes that the timid water saw its Lord and blushed is prepared to accept the later miracles effected by Skunk cabbage or some alliterative nostrum. It would seem that the reverse English could be put on the simple of faith. It is certainly a very undignified position for the followers of a learned profession to be set up as the most gullible class of society. Some ministers of the gospel are so forgetful of their ghostly calling as to lend not only their indorsement but their dollars to aid in marketing some nostrum. Such instances of the degradation of a high calling are fortunately rare.

The editor of a religious publication should always hold in mind the immense influence wielded by his paper in the family circle, and remember before he admits questionable advertising matter into his columns that to many of his readers such advertisements are treated as though they bore the stamp of editorial recommendation and approval, and that the inevitable discovery of the false asertions as to the merits of the remedy will react upon the paper itself. There are enough men advertising church furniture, bells and books to keep the paper alive even if the rural shepherd is slow with his subscription.

---

DR. ALAN P. EBBERT died of pneumonia at his residence on Capitol avenue, November 28. Dr. Ebbert was a veteran of the Spanish-American war and was prominent in fraternal orders.

---

A BAZAAR will shortly be held for the purpose of raising funds for a maternity ward at the Grady Hospital. The limited facilities at the hospital for such work would make this addition very desirable.

